# Emergence of Epizootic Ulcerative Syndrome in Native Fish of the Murray-Darling River System, Australia: Hosts, Distribution and Possible Vectors

**DOI:** 10.1371/journal.pone.0035568

**Published:** 2012-04-25

**Authors:** Craig A. Boys, Stuart J. Rowland, Melinda Gabor, Les Gabor, Ian B. Marsh, Steven Hum, Richard B. Callinan

**Affiliations:** 1 New South Wales Department of Primary Industries, Port Stephens Fisheries Institute, Nelson Bay, New South Wales, Australia; 2 New South Wales Department of Primary Industries, Grafton Fisheries Centre, Grafton, New South Wales, Australia; 3 State Veterinary Diagnostic Laboratory, NSW Department of Primary Industries, Elizabeth Macarthur Agricultural Institute, Menangle, Narellan, New South Wales, Australia; 4 Faculty of Veterinary Science, University of Sydney, Camden, New South Wales, Australia; Smithsonian's National Zoological Park, United States of America

## Abstract

Epizootic ulcerative syndrome (EUS) is a fish disease of international significance and reportable to the Office International des Epizootics. In June 2010, bony herring *Nematalosa erebi*, golden perch *Macquaria ambigua*, Murray cod *Maccullochella peelii* and spangled perch *Leiopotherapon unicolor* with severe ulcers were sampled from the Murray-Darling River System (MDRS) between Bourke and Brewarrina, New South Wales Australia. Histopathology and polymerase chain reaction identified the fungus-like oomycete *Aphanomyces invadans*, the causative agent of EUS. Apart from one previous record in *N. erebi*, EUS has been recorded in the wild only from coastal drainages in Australia. This study is the first published account of *A. invadans* in the wild fish populations of the MDRS, and is the first confirmed record of EUS in *M. ambigua*, *M. peelii* and *L. unicolor*. Ulcerated carp *Cyprinus carpio* collected at the time of the same epizootic were not found to be infected by EUS, supporting previous accounts of resistance against the disease by this species. The lack of previous clinical evidence, the large number of new hosts (n = 3), the geographic extent (200 km) of this epizootic, the severity of ulceration and apparent high pathogenicity suggest a relatively recent invasion by *A. invadans*. The epizootic and associated environmental factors are documented and discussed within the context of possible vectors for its entry into the MDRS and recommendations regarding continued surveillance, research and biosecurity are made.

## Introduction

The emergence and spread of aquatic freshwater diseases are a major conservation concern [1]. One aquatic disease implicated in mass mortalities of cultured and wild fish in many countries is epizootic ulcerative syndrome (EUS) [2]. Also known as mycotic granulomatosis, red spot disease and ulcerative mycosis, EUS is caused by the fungus-like oomycete *Aphanomyces invadans* ( = *A. piscicida*) and can cause significant ulceration of the skin, necrosis of muscle with extension to subjacent structures including abdominal cavity and cranium, and leading to mortality in many cases [2]–[4]. Originally described in cultured ayu *Plecoglossus altivelis* in Japan [5], within three decades EUS had been reported in more than 100 fish species [6] in both freshwater and estuarine environments throughout south, south-eastern and western Asia [7]–[9], the east coast of North America [10], [11], in distinct regions of Australia: including New South Wales (NSW), Northern Territory, Queensland and Western Australia [3] and recently in Africa [12]. Due to concern over its potential impact on cultured and wild fisheries, EUS is officially recognised as a reportable disease by the Network of Aquaculture Centres in Asia-Pacific (NACA) and internationally by the World Organisation for Animal Health (Office International des Epizooties or OIE) [13].

Little is known about the infectious diseases of native fish in the Murray-Darling River System (MDRS), which drains inland catchments, west of the Great Dividing Range in south-eastern Australia. Until recently (2001 in an aquaculture facility), EUS had not been reported from the MDRS, and within Australia was generally considered endemic only to coastal drainages. Originally referred to as Bundaberg Fish Disease, EUS was first reported in Australia in 1972 [14], and subsequently there have been numerous outbreaks reported in wild freshwater and estuarine fishes in the eastern, northern and western coastal drainages [15]–[18].

EUS has been reported in silver perch *Bidyanus bidyanus* (Mitchell, 1838), a species endemic to the MDRS, being farmed in coastal drainages in northern NSW and south-eastern Queensland [19]. More recently, there have been reports of the presence of EUS within the MDRS, although little is known of its pathogenicity, distribution or susceptibility of species to infection in wild populations in this river system. In May 2008, *A. invadans* was isolated from bony herring *Nematalosa erebi* (Günther, 1868) immediately upstream of Bourke town weir on the Barwon-Darling River, representing the first confirmed, reported diagnosis of EUS in the MDRS, and significantly extending the known range of this pathogen within Australia (J. Go, Elizabeth Macarthur Agricultural Institute, unpubl. data).

During routine sampling of fish in the same section of the Barwon-Darling River in June 2010, dermal lesions and ulcers were observed in six species of fish. Many of the lesions and ulcers appeared to be characteristic of EUS, raising the concern of an epizootic of this reportable disease. As a member country of the OIE, Australia is obliged to report any significant new incursions of EUS, whether it is in a new species or a new area. This paper is the first published account of EUS in four native species: bony herring, golden perch *Macquaria ambigua* (Richardson, 1845), Murray cod *M. peelii* and spangled perch *Leiopotherapon unicolor* (Günther, 1859). The objectives are three-fold. Firstly to document the 2010 epizootic and report its prevalence and extent compared to the time of its first report in *N. erebi* in 2008. Secondly, to document some of the environmental factors associated with the epizootic. Finally, potential explanations of the route of introduction of *A. invadans* into the MDRS are suggested and discussed, with recommendations made for future surveillance, research and biosecurity.

## Results

### Diagnosis of EUS

**Figure 1:**
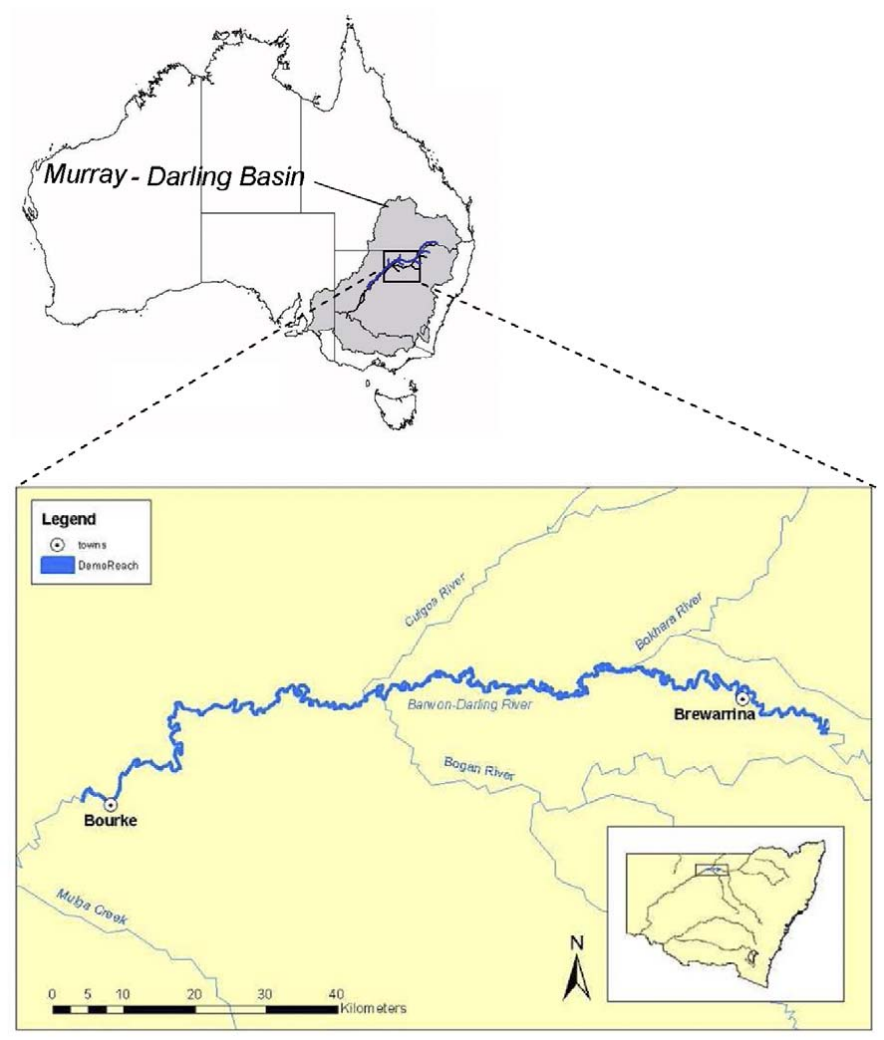
Section of the Barwon-Darling River between Bourke and Brewarrina weirs where fish with EUS were collected.

**Figure 2:**
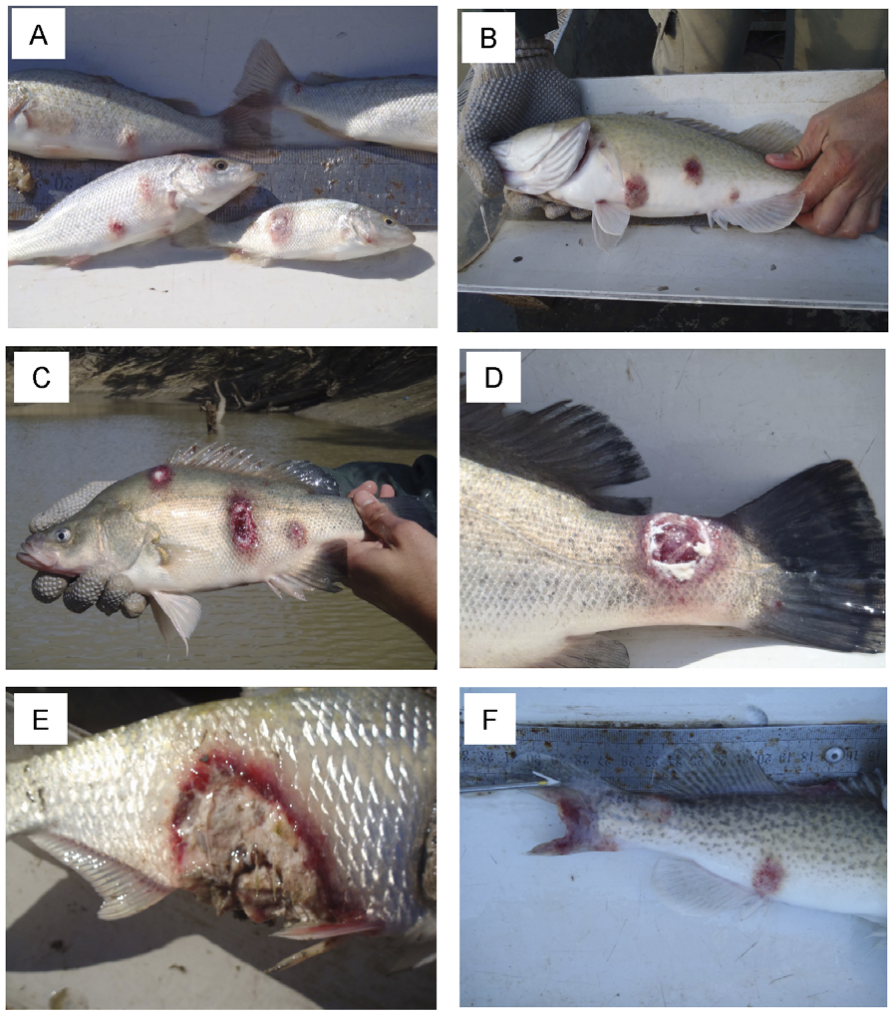
Diseased fish collected from the Barwon-Darling River between Bourke and Brewarrina weirs in June 2010. Panels show A) *L. unicolor*, B) *M. peelii* with raised lesions, C,D) *M. ambigua* and F) *M. peelii* with deep ulceration and muscle or fin necrosis, and E) *N erebi* showing severe ulceration and tissue necrosis exposing the peritoneal cavity and internal organs.

In June 2010, *N. erebi*, *M. ambigua*, *M. peelii*, *L. unicolor*, carp *C. carpio* and goldfish *C. auratus* caught from a 200 km section of the Barwon-Darling River between Bourke and Brewarrina weirs (Figure 1) had raised lesions and mild to severe ulcers characteristic of the invasive, tissue-destructive stages of EUS (Figure 2A–F). In some fish the caudal peduncle, caudal fin or dorsal fins were severely eroded (Figure 2D,F), and in others, deep ulcers penetrated into and exposed the peritoneal cavity (Figure 2E).

*Aphanomyces invadans* was detect as being present in *N. erebi*, *M. ambigua*, *M. peelii* and *L. unicolor* using histopathology, and the diagnosis was further confirmed in *N. erebi* and *M. ambigua* using PCR. Although a small number of *C. carpio* and *C. auratus* were sampled with distinct haemorrhagic, dermal lesions, histopathology and PCR (PCR performed on *C. carpio* only) confirmed that these were not consistent with the case definition of *A. invadans* [2], [6], [20]. PCR was not performed on *L. unicolor* and *M. peelii*.

**Figure 3:**
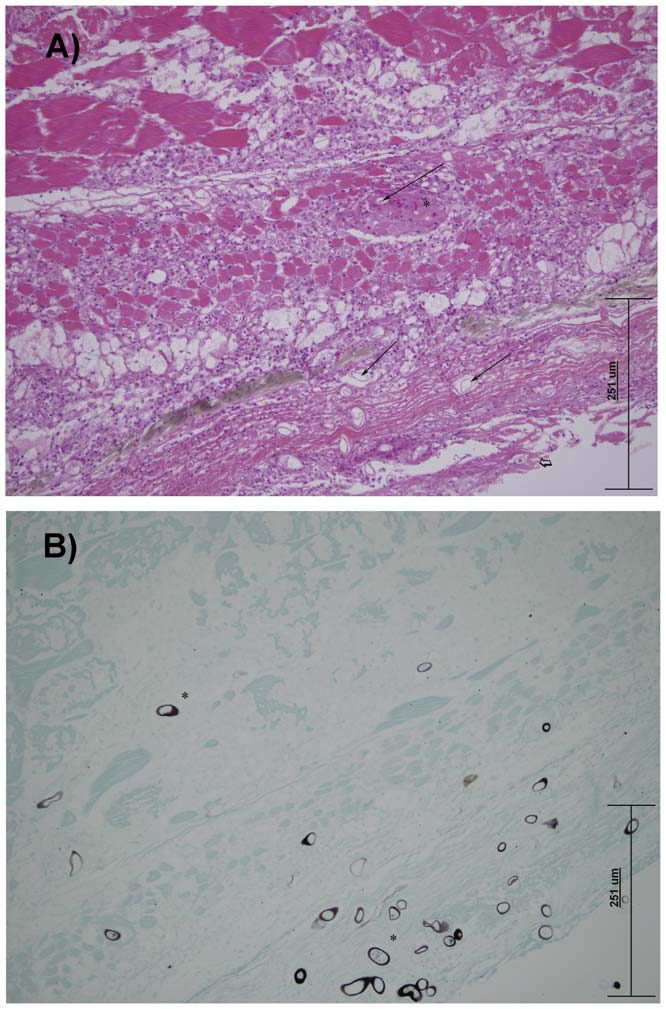
A) *N. erebi*, skin and underlying muscle. Photo micrograph of developing linear granulomas (thin arrow) surrounding faintly eosinophilic fungal hyphae (*). The overlying epithelium is ulcerated (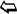) H & E. (X200). B) *N. erebri*, skin and underlying muscle. Photo micrograph of black staining longitudinal and cross sectional fungal hyphae (*) against green stained tissue. GMS. (X200).

Gross observations were consistent in all of the confirmed cases, with focal to multifocal cutaneous ulceration of varying degrees of severity and distribution. Histologically, there was extensive necrosis and ulceration of the epidermis with adjacent epithelial hyperplasia. Subjacent myofibres were, in most cases, severely necrotic, with extensive myofibrillar liquefaction. The endomysium was infiltrated with moderate numbers of histiocytes, lymphocytes and plasma cells, with lesser granulocytes. Distinct, thin sheaths of macrophages (linear granulomas) were frequently observed, surrounded by a narrow band of lymphocytes and plasma cells (Figure 3A). In all cases, myriad fungal hyphae were associated with the ulcers, infiltrating into the myofibres and surrounding connective tissue. Cross sectioned hyphae varied from 10–35 µm in width, non-septate, thick-walled, with occasional branching. The morphology of hyphae was accentuated with GMS staining (Figure 3B). Typically, abundant fibrinocellular debris was associated with the eroded surfaces. In some *L. unicolor* and *N. erebi*, there were multiple infiltrative granulomas associated with fungal hyphae within internal organs, including kidneys, abdominal adipose tissue, ovary and swim bladder.

### Nucleic acid detection of A. invadans

PCR performed on tissue samples detected *A. invadans* DNA in three of three *N. erebi* and three of four *M. ambigua* samples. A negative PCR result was obtained for one *C. carpio* analysed.

### Difference in the prevalence of ulcerated fish between 2008 and 2010

**Figure 4:**
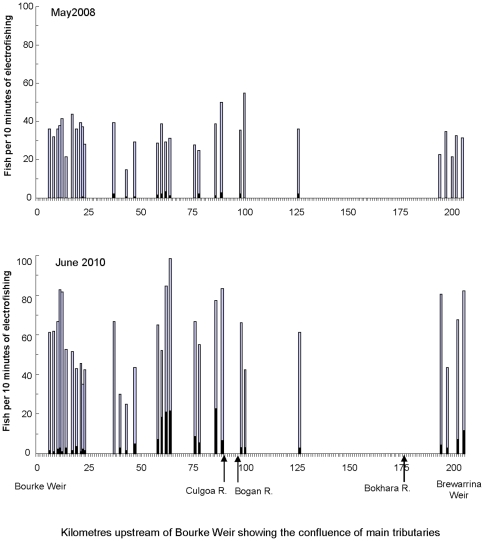
Distribution of fish in the Barwon-Darling River between Bourke and Brewarrina weirs with ulcers (black) and without ulcers (grey) in 2008 and 2010.

**Figure 5:**
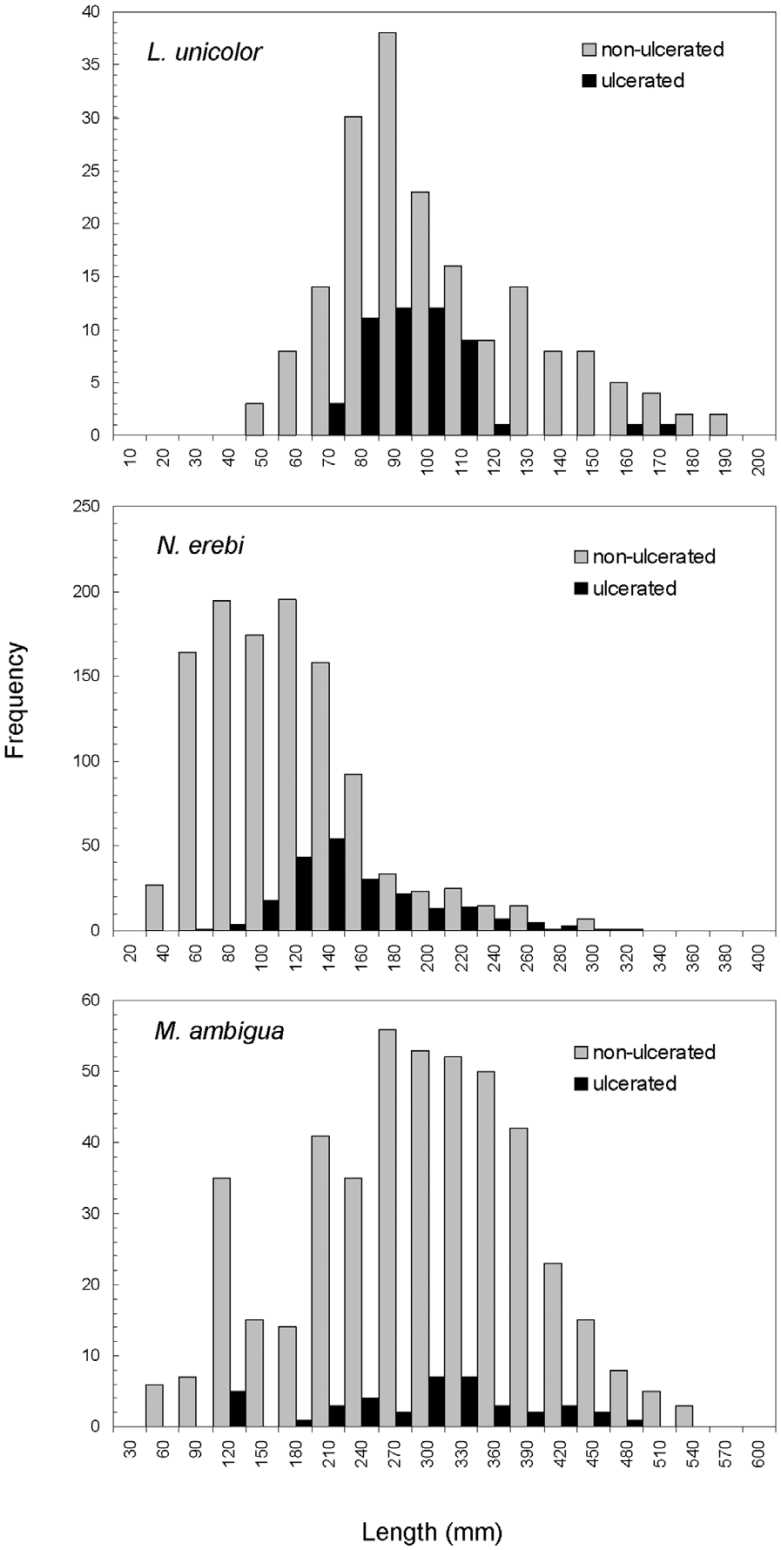
Length frequency of ulcerated and non-ulcerated fish collected from the Barwon-Darling River between Bourke and Brewarrina weirs in June 2010.

In 2008, ulcerated fish representing four species were sampled at 13 of the 30 locations over 200 km from Bourke Weir to Brewarrina Weir, although only N. erebi were submitted for histopathology testing and subsequently confirmed to have EUS (Jeffery Go, unpublished data). By comparison, in 2010 six species with ulcers were sampled from 29 of the 30 locations (Table 1; Figure 4). The prevalence of cutaneous lesions and/or ulcers was 2% (of all fish sampled) in 2008, but 10% in 2010 (Table 1). No *N. erebi*, *L. unicolor* and *M. ambigua* <60 mm had lesions or ulcers (Figure 5). Ulcerated *L. unicolor* ranged in length from 70–170 mm (n = 50), *N. erebi* 60–320 mm (n = 216), and *M. ambigua* 120–480 mm (n = 40). Most ulcerated *L. unicolor* were in the range 70–130 mm, and a majority of *N. erebi* over 140 mm were ulcerated (Figure 5).

**Table 1:** Number of ulcerated fish from the assemblage of species collected from 30 locations in the Barwon-Darling River between Bourke and Brewarrina weirs in May 2008 and June 2010.

Common name	Scientific name	May 2008	June 2010
Olive perchlet	*Ambassis agassizii* (Steindachner, 1866)	-	-
Silver perch	*Bidyanus bidyanus*	-	-
goldfish	*Carassius auratus*	-	2 (<1%)
Un-specked hardyhead	*Craterocephalus stercusmuscarum fulvus* (Ivantsoff, Crowley & Allen, 1987)	-	-
Carp	*Cyprinus carpio*	7 (3%)	7 (<1%)
Mosquito fish	*Gambusia holbrooki* (Girard, 1859)	-	-
Carp gudgeon	*Hypseleotris* spp.	-	-
**Spangled perch**	***Leiopotherapon unicolor***	3 (8%)	**50 (21%)**
**Golden perch**	***Macquaria ambigua***	3 (2%)	**40 (8%)**
**Murray cod**	***Maccullochella peelii***	-	**4 (5%)**
Murray-Darling rainbowfish	*Melanotaenia fluviatilis* (Castelnau, 1878)	-	-
**Bony herring**	***Nematalosa erebi***	**28 (<1%)**	**216 (16%)**
Hyrtl's tandan	*Neosiluris hyrtlii* (Steindachner, 1867)	-	-
Australian smelt	*Retropinna semoni* (Weber, 1895)	-	-
Total number individuals ulcerated		41 (2%)	319 (10%)
Total number of species ulcerated		4 (29%)	6 (32%)

Data in parentheses are the proportion of sampled individuals of each species that were ulcerated. Bold type identifies species in which EUS was confirmed using histopathology. No data means that the species was caught but no specimens were ulcerated.

### Environmental conditions

**Figure 6:**
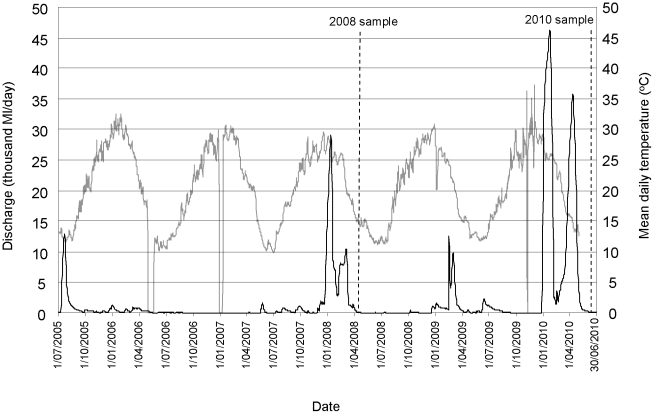
Daily discharge (black line) and mean daily water temperature (grey line) recorded at the Bourke gauge (425003) between July 2005 and July 2010; dotted lines show when the 2008 and 2010 fish samples were collected. Source: NSW Office of Water.

The epizootics occurred in autumn (2008) and winter (2010) with water temperatures below 16°C and decreasing (Figure 6). In both years, detection of ulcerated fish occurred within two months of significant within-channel flow events (equivalent to between four and eight percentile flows at the Bourke gauge), after an extended period of low-flow conditions (Figure 6). Water quality variables monitored at the time of fish sampling (i.e. once lesions/ulcers were already established) were: temperature 12.7–16.3°C; pH 7.3–8.7; electrical conductivity 350–600 µs.cm^−1^ (electrical conductivity was not significantly different from the five year average obtained from the Bourke gauge; Student *t*-test, d.f. 79, p = 0.593); and dissolved oxygen 4.2–10.6 mg.L^−1^. Dissolved oxygen concentrations were not below acceptable trigger values for aquatic ecosystems for lowland rivers in south eastern Austrlaia [21].

## Discussion

### Hosts and distribution

Gross observations and histopathology identified EUS as the cause of the recent epizootic in the Barwon-Darling River in 2010. This is the first published account of EUS in *N. erebi* and first confirmed case of the disease in the native species *M. ambigua, M. peelii* and *L unicolor* in the wild. The findings are consistent with the earlier unpublished reports of EUS in *N. erebi* in the Barwon-Darling River in 2008 (Jeffery Go, unpublished data). This increases the number of native fish in the MDRS known to be susceptible to EUS to five, with *B. bidyanus* previously being shown to carry the disease under some culture conditions [19]. Additionally, there has recently (January 2010) been unpublished confirmation of EUS in farmed *M. peelii* at a facility on the Murray River (Brett Ingram and Tracey Bradley, pers. comm). Prior to this, the disease had never been reported in wild or cultured *M. peelii* [22].

*Carasius auratus* are known to be susceptible to EUS [23], however, we found no histopathological evidence that *A. invadans* was the causative pathogen in the two ulcerated *C. auratus* sampled during the recent epizootic. Similarly, despite the abundance of *C. carpio* (n = 953) sampled in the study area during the epizootic, and the observation of seven ulcerated individuals, there was similarly no evidence that *A. invadans* was the causative pathogen for the individuals examined. This is consistent with current evidence through Asia and Europe that *C. carpio* is resistant to EUS [6], [24], [25].

Prior to 2008, all previous reports of EUS in Australia were from coastal drainages from Bundaberg in Queensland to the Hawkesbury River in NSW [6], [14], [16], [18], [19], [26]. Following the first report of red spot disease in Australia near Bundaberg in Queensland in 1972 [14], numerous outbreaks followed during the 1980s, and the distribution of the disease expanded south to include the Clarence and Richmond rivers in NSW [16], [18], [26]. Elsewhere in the world, EUS has spread rapidly throughout south-east Asia and extended deep into the Indian sub-continent since the mid 1980s [27]. These and more recent reports from Africa, Zambia and the USA [12], [28]–[30] demonstrate the extent and ease at/with which this disease can spread.

EUS is a very invasive disease, and when it first occurs in an area, there are high levels of mortality over a very short time, many susceptible species are affected, and individual fish can have numerous lesions and ulcers [27], [31]. The severity and prevalence of this recent epizootic and lack of previous reporting suggests that EUS may be a recent incursion into the MDRS. More than 100 fish species have been reported to be affected by EUS [6], and susceptibility varies between species, with some, including tilapia *Oreochromis* spp. (Castelnau, 1861) resistant to infection by *A. invadans* [24], [25], [32], [33]. Resistance to EUS in tilapia is of significance to the Murray-Darling River System as this species currently poses a significant invasion risk in northern catchments [34]. The severity of ulceration that we observed in *N. erebi*, *M. ambigua* and *M. peelii* suggest that these species are very sensitive to infection by *A. invadans*.

The current geographic distribution of EUS in the MDRS remains largely unclear. The epizootic reported in this study covers a 200 km section of the Barwon-Darling River in the northern region of the Murray-Darling Basin. During publication of this paper, EUS has also been confirmed (histopathologically) in two *M. ambigua* sampled (in July 2011) below Lock 7 in the Murray River, a site downstream of the confluence with the Darling River (M. Gabor and D. Gilligan, NSW Department of Primary Industries, unpubl. data). This, and the previously mentioned report of infected *M. peelii* on a fish farm near the Murray River, confirms that this disease is now widespread in the Murray-Darling River system.

### Predisposing environmental factors

Exposure to *A. invadans* spores is a key factor causing EUS [2], and the incidence and transmission of this pathogen throughout the MDRS will largely determine the potential range this disease. Once a pathogen is in an area, subsequent outbreaks of infectious diseases in fishes are closely linked to environmental conditions, particularly temperature and other water quality variables through their effects on stress and the immune system [35]–[37]. The prevalence of EUS in four species in the Barwon-Darling River in 2010 suggests that conditions were conducive to initial infection and transmission of the pathogen within and between species [2]. Caution must be exercised when interpreting the significance of the water quality measurements taken during this study, as they were taken only at the time of sampling, when fish were ulcerated and the epizootic well advanced. Nevertheless, it is prudent to document the environmental variables associated with all epizootics, to facilitate the development of causal links should more data become available from future outbreaks.

Both outbreaks in 2008 and 2010 were detected at relatively low water temperatures (<16.3°C) following periods of very high flow and flooding. This is consistent with most outbreaks of EUS, which tend to be associated with low and declining water temperatures and high rainfall [6], [9], [25], [33]. Virgona [16] reported significant correlation between rainfall and the prevalence of early stage lesions in sea mullet *Mugil cephalus* (L., 1758), and found that progression to later stage ulcers occurred after the high flows. Outbreaks of EUS in cultured *B. bidyanus* at the Grafton Aquaculture Centre occur only when water is pumped from the Clarence River during high flows or floods, and when fish in the river are known to have EUS [38].

Temperature is a critical factor determining the severity of EUS outbreaks and most mortalities occur when water temperatures are relatively low [39]. The findings in this paper suggest that high flows and low temperatures may have been predisposing factors to the outbreaks in 2008 and 2010. Low water temperatures (<16°C) and rapid decreases in temperature are immunosuppressive and induce changes to the epidermis, including loss of mucus that predispose fish to infection [37], [40]. Outbreaks of the fungal disease saprolegniosis during winter can cause significant mortalities in many species of freshwater fish in the wild and under culture conditions, including *N. erebi* and *B. bidyanus* [38], [41].

Other water quality variables including low pH, low dissolved oxygen, decreasing alkalinity, hardness and conductivity have been implicated in outbreaks of EUS [6], [9], [33]. Although low pH (<6) has been associated with some EUS outbreaks [12], [17], Rowland [42] reported an outbreak of EUS in *B. bidyanus* in earthen ponds following a bloom of the blue-green algae *Microcystis* and a rapid rise in afternoon pH values to 9.4 and unionised ammonia to 0.39 mg/L. These findings suggest that rapid changes in pH and possibly other water quality variables, and not necessarily absolute values, may initiate changes to the skin which allow attachment of *A. invadans* spores and subsequent invasion of underlying tissue as suggested by Callinan *et al.* [18]. Outbreaks of EUS in the Richmond River in Australia, as well as in the Philippines, Bangladesh and Zambia have been attributed to exposure to acidic runoff draining from acid sulphate soils following heavy rainfall [9], [12], [43], [44]. Sulphidic sediments are not uncommon in floodplain wetlands of the MDRS, potentially being caused by hydrological change brought about by river regulation [45]. Although the risk of wetland acidification appears to be lowest in the Darling River within the vicinity of the recent EUS outbreak, areas of the Murray River appear to contain sulfidic sediments at concentrations which could pose an acidification risk [45]. It is plausible that recent record low flows in the MDRS, combined with the reinstatement of wetting and drying regimes to wetlands may alter pH and provide conditions that predispose fish to infection by *A. invadans*. Extensive cyanobacterial blooms are known to occur in the Barwon-Darling River [46], but there were no reports coinciding with either of the epizootics.

### Possible vectors for introduction of EUS into the MDRS

Controlling the spread of infectious diseases through cultured fish has been a serious problem in many countries, including Australia [47], [48]. It is unclear how *A. invadans* has entered the MDRS, but the translocation of cultured *B. bidyanus*, *M. peelii* and *M. ambigua* may have provided a vector for its introduction. In the past, fingerlings have been translocated from Government and commercial native fish farms in eastern drainages in southern Queensland and north-eastern NSW to the western drainage for stock enhancement in impoundments and for commercial aquaculture. In 2001, *B. bidyanus* with EUS were translocated from a commercial hatchery in north-eastern NSW to a fish farm on the Murray River, and although quarantine procedures were implemented, it is unsure if pathogens escaped from the farm (R. Callinan and S. Rowland, unpubl. data). A hatchery quality assurance program and biosecurity measures in NSW now prevent the translocation of native fish fingerlings from eastern drainages to the MDRS, and all fingerlings leaving each hatchery must be free of pathogens and signs of diseases [49]H. Such measures should now be considered for all within-MDRS translocations, including the interstate movement of fish. Until the aspects of distribution and hosts of EUS are better known, no fish should be transported from the Barwon-Darling River to hatcheries for use in breeding programs unless they can be certified free of pathogens.

The extensive, east to west migration of waterbirds from coastal drainages may be a potential vector for the translocation of *A. invadans* to the MDRS that warrants further investigation. Waterbirds are known to disperse a range of aquatic organisms [50]H, and fish-eating birds have been implicated in the spread of some infectious diseases in fish [51]–[53]. Cormorants *Phalacrocorax* spp., pelicans *Pelecanus conspicillatus*, ibis *Threskiornis* spp., and various species of ducks, including grey teal *Anas gracilis* are commonly found in both coastal and inland drainages, at times aggregating in large numbers on *B. bidyanus* farms where they can introduce pathogens from the wild and move pathogens from pond to pond [38], [42]; Jeff Guy, pers. comm.).

It is plausible that *A. invadans* may have been carried into the MDRS from coastal drainages in boats or other equipment. Boats and fishing equipment have been implicated in the transportation of larval and adult stages of some aquatic organisms (in live wells, bilges, bait buckets and engines and the transmission of whirling disease in trout in the USA [54]–[56]. Whilst there is no evidence that this is responsible for the movement of *A invadans* into the MDRS, it cannot be discounted because is not uncommon for research boats involved in the State and Basin-wide monitoring programs to frequently move between coastal and inland drainages. It would therefore be prudent to ‘disinfect’ boats and equipment moving between different aquatic environments and this warrants further consideration by biosecurity agencies.

### Conclusions

The five known host native fish species, *N. erebi*, *M. ambigua*, *M. peelii*, *L. unicolor* and *B. bidyanus* appear very susceptible to infection by *A. invadans*. Given the invasive nature of EUS, it can be expected to spread to other parts of the MDRS, and the recent confirmation of EUS in farmed and wild fish in the Murray River demonstrates that the disease in not restricted to the Barwon-Darling River. Although no attempt was made to estimate the level of mortality in our study, EUS is known to cause losses of 100% in susceptible species in captivity [29], [57]. Oomycete infections cause significant problems in aquaculture and have been implicated in the decline of some wild fish stocks around the world [58]. In *B. bidyanus* culture, the prevalence of EUS can be as high as 90%, with mortality rates >50% in tanks, and winter saprolegniosis can cause total mortality of *B. bidyanus* in earthen ponds [38], [59]. The invasive nature of EUS and apparent pathogenicity to at least five endemic species suggest that it poses a significant threat to the fishes of the MDRS warranting surveillance, more vigilant reporting, pathology testing of suspected outbreaks and further research into its potential spread and impact on wild an cultured fish. This will assist in the development of sound biosecurity and fisheries management actions to combat this emerging disease.

## Materials and Methods

### Ethics statement

All field studies outlined in this paper were authorised under a scientific research permit (permit No: P01/0059) issued by the NSW Department of Primary Industries under section 37 of the Fisheries Management Act 1994. This permit authorises the collection of fish in all waters of NSW. The river sites sampled were not privately owned or protected and no endangered or protected species were involved in this study. All fish collection was carried out in an ethical manner and any fish euthanased were done so in accordance with the Australian code of practice for the care and use of animals for scientific purposes (2004) and NSW Primary Industries (Fisheries) Animal Research Authority 98/14.

### Fish and water sampling

Fish were sampled from 30 locations in the Barwon-Darling River over approximately 200 km of river channel between the town weirs of Bourke (30°05′12.77″S, 145°53′39.41″E) and Brewarrina (29°57′27.63″S, 146°51″24.73″E) in north-western NSW (Figure 1). At each location, fish were immobilised using a total of 1080 seconds (‘power-on’) of electric fishing (boat-mounted 7.5 kW Smith-Root Model GPP 7.5 H.L^−1^). All fish were collected using a 3 mm mesh dip net and placed in a live-well before being identified, measured (fork length (FL) for fork-tailed species and total length (TL) for others) and assessed for disease or abnormalities. The total catch and the number of individuals and species with lesions or ulcers were compared to data obtained using equivalent methods and fishing effort from the same 30 locations in 2008 (obtained from the Department of Industry & Investment NSW, Freshwater Fish Research Database). Dissolved oxygen, pH, temperature and conductivity were recorded at the time of sampling using a Horiba U-10 water quality meter near the surface (<1 m) for each location in 2010.

### Histopathology

Diagnostic tests for EUS were carried out on a sub-sample of ulcerated fish (haphazardly selected). After capture, these fish were immediately sealed in bags (one fish per bag) and placed in an ice slurry to induce euthanasia and to preserve specimens until a fixative could be added. Specimens (n = 23) sent for laboratory examination were *L. unicolor* (n = 2), *N. erebi* (n = 7), *M. ambigua* (9), *M. peelii* (n = 1), carp *Cyprinus carpio* (Linnaeus, 1758) (n = 2) and goldfish *Carassius auratus*, (L., 1758) (n = 2).

We used a case definition currently accepted by OIE to confirm the presence of EUS by histopathology. This involved identifying mycotic granulomas in histological sections, with further isolation of *A. invadans* from internal tissues in a subset of cases (Level II diagnosis: [2], [6], [20]. Within 24 to 48 hours, necropsy examination and sample fixing was carried out on all submitted fish. Samples of skin, underlying muscle tissue and major internal organs were removed and fixed in 10% neutral buffered formalin, and processed for histological evaluation in a standard manner. Slides were stained with Haematoxylin and Eosin (H&E) and Gomoris Hexamine Silver (GMS: [60]). Additionally, samples of tissues underlying ulcers were fixed in 95% ethanol prior to molecular examination.

### Nucleic acid detection of A. invadans

DNA extraction from ulcerated lesion of examined fish and EUS PCR was performed as described by Buller *et al.* [61] and the manufacturers recommendations for DNAzol. Briefly, 25–50 mg tissue (about 5 mm) was homogenised in 700 µL of DNAzol reagent (DNAzol® Genomic DNA Isolation Reagent, Molecular Research Centre Inc., Cat. No. DN 127). The homogenate was allowed to stand at room temperature for five to 10 minutes and then centrifuged for 10 minutes at 16,060×*g*. The supernatant was transferred to a fresh tube and the DNA was precipitated by adding 400 µL of 100% ethanol and mixing by inversion. This was allowed to stand at room temperature for 1 minute and then centrifuged for five minutes at 3421×*g*. The supernatant was removed and the pellet was washed twice with 600 µL of 75% ethanol by inverting the tube three to six times to re-suspend the DNA then centrifuging for three minutes at 3421×*g* to collect the DNA. The remaining ethanol was removed by pipette and the DNA was air dried for five to 15 seconds. The DNA pellet was dissolved by 8 mM NaOH (pellet <2 mm diameter: 50 to100 µL, pellet >2 mm diameter: 150 µL) and then stored at 4°C for immediate use or −20°C for long term storage.

A specific PCR for the direct detection of *A. invadans* in tissue, that targets a 554 bp region of the internal transcribed spacer (ITS) regions [61], [62], was undertaken in a 25 µL reaction including: 12.5 µL of Promega PCR Master Mix (Promega Cat. No. M7502), 0.5 µL (800 nM) of each primer (AIFP10, ATTACACTATCTCACTCCGC and AIFP 14, CTGACTCACACTCGGCTAGC), 2.0 µL of template DNA and purified water. Amplification was then performed in a 96-place thermal cycler (Corbett Research, Sydney, Australia) using the following conditions: one cycle of denaturation at 94°C for five minutes followed by 35 cycles of denaturation at 94°C for one minute, annealing at 55°C for 30 seconds, extension at 72°C for 30 seconds and a final extension of one cycle at 72°C for five minutes. PCR results were assessed by electrophoresis in 2% agarose gels stained with ethidium bromide. A sample was considered positive when a 554 bp product was produced corresponding to the *A. invadans* positive control.
